# Correlation Between Apparent Diffusion Coefficient Value on MRI and Histopathologic WHO Grades of Neuroendocrine Tumors

**DOI:** 10.5334/jbsr.1925

**Published:** 2020-01-30

**Authors:** Wouter Mebis, Annemiek Snoeckx, Bob Corthouts, Haroun El Addouli, Simon Nicolay, Astrid Van Hoyweghen, Maarten Spinhoven, Bart Op de Beeck

**Affiliations:** 1 University Hospital Antwerp, BE

**Keywords:** apparent diffusion coefficient, magnetic resonance imaging, neuroendocrine tumor, histopathological grade, quantitative

## Abstract

**Background:**

The correlation of diffusion-weighted MRI and tumor aggressiveness has been established for different tumor types, which leads to the question if it could also apply for neuroendocrine tumors (NET).

**Purpose:**

To investigate the possible correlation between apparent diffusion coefficient (ADC) value on magnetic resonance imaging (MRI) and histopathologic WHO-grades of NET.

**Material and Methods:**

Electronic patient records from patients presented at the multidisciplinary neuro-endocrine tumor board between November 2017 and April 2019 were retrospectively reviewed. Patients with both available MR imaging (primary tumor or metastasis) and known WHO tumor grade were included (n = 47). Average and minimum ADC values (avgADC; minADC) were measured by drawing a freehand ROI excluding only the outermost border of the lesion. The largest axial size (primary tumor) or most clearly delineated lesion (metastasis) was used.

**Results:**

Forty seven patients met the inclusion criteria (mean age 59 ± 12 SD; 24F/23M). Twenty one patients (45%) were diagnosed with WHO G1 tumor, 17 seventeen with G2 (36%) and nine with G3 (19%) tumor. Twenty eight primary tumors and 19 metastases were measured. A significant difference was found between low-grade (G1+G2) and high-grade (G3) tumors (Mann-Whitney; avgADC: p < 0,001; minADC: p = 0,001). There was a moderate negative correlation between WHO-grade and avgADC/minADC (Spearman; avgADC: –0,606; 95% CI [–0,773; –0,384]; minADC: –0,581; 95% CI [–0.759; –0.353]).

**Conclusion:**

Our data show a significant difference in both average and minimum ADC values on MRI between low and high grade NET. A moderate negative correlation was found between histopathologic WHO grade and ADC value.

## Introduction

Neuroendocrine tumors (NET) are derived from neural crest cells that are diffusely distributed throughout the human body. This explains the various primary NET locations including lung/bronchus, pancreas, small intestine, colon and rectum. NET are relatively rare, accounting for 0.46% of gastrointestinal, pancreatic and lung malignancies [1]. The incidence and prevalence of NET has increased over time due to increased diagnosis and better survival respectively. Data from the United States Surveillance, Epidemiology, and End Results (SEER) program indicate a NET incidence of 6.98 per 100,000 [2]. NET can be subdivided according to their functional activity (based on the production of hormones) or histopathological grade. Functional NET are often detected in a relatively early stage due to the symptomatology related to production of hormones. Non-functional NET are more often found incidentally or remain undetected until a later stage when symptoms arise from locoregional mass effect or distant metastases. The natural disease progression, therapeutic response, and survival varies among different primary tumor locations, functional state, and more importantly, histopathological grade [1,2,3].

Different histopathological grading systems exist, with the European Neuroendocrine Tumor Society (ENETS) and World Health Organization (WHO) criteria being the most widely accepted. In this study we used the recently revised WHO 2017 grading system for pancreatic NET and the WHO 2010 grading system for all other NET. The WHO grading system is based on the Ki67 and mitotic indices to classify NET into low (G1), intermediate (G2), and high grade (G3) tumors [4].

The Ki67-index is a proliferation index based on the presence of the Ki-67 cellular marker in proliferating cells. Its presence can be demonstrated by immunostaining with monoclonal anti-Ki-67 antibodies. The percentage of Ki-67 positive cells is determined in tumor hot spots where a minimum of 500 cells is counted. The mitotic index is the number of mitoses counted per high power field (HPF). Generally, mitoses are counted in 50 HPF and the mitotic index is expressed in mitoses per 10 HPF [5].

The 2017 update for pancreatic NET altered the cut-off value for NET G1 and added a subclassification of G3 tumors dividing them into well-differentiated G3 NET and poorly-differentiated G3 neuroendocrine carcinomas (NEC). The different cut-off values are demonstrated in Table 1 [4].

**Table 1:** WHO Classification for Neuroendocrine Neoplasms (2010–2017).

Grade	Ki67-index (%)		Mitotic index (mitoses/10 HPF)		Differentiation

WHO	2010	2017 (pNET)	2010	2017 (pNET)	
NET G1	≤2	<3	<2		Well differentiated
NET G2	3–20	3–20	2–20		Well differentiated
NET G3	>20	>20	>20	>20	Well differentiated
NEC G3		>20		>20	Poorly differentiated (small/large cell)

The most notable differences of the 2010 and 2017 World Health Organization (WHO) classification system for NET is the increase of the Ki67-index cut-off value for G1 NET to <3 and the differentiation between well differentiated G3 NET and poorly differentiated G3 neuroendocrine carcinomas (NEC).

The diagnosis and characterization of NET is based on both laboratory testing with serum markers such as Chromogranin A (and specific hormone levels for functional NET) and multimodality imaging. Different imaging techniques are available including ultrasound (US), computed tomography (CT), magnetic resonance imaging (MRI) and functional/nuclear imaging such as somatostatin receptor imaging and positron emission tomography (PET). The combination of PET and CT (PET/CT) with different tracers can be especially valuable in NET staging and detection of metastases. Fluorine 18 fluorodeoxyglucose (FDG) PET/CT tracer is widely used in oncologic imaging but appears to be of limited value in well-differentiated NET because of the near normal glucose turnover. NET that do not show a high uptake on ^18^F-FDG-PET, can be investigated with a number of somatostatin analogs labelled with Gallium 68 (^68^Ga) (i.e. ^68^Ga-DOTA-NOC) which bind to the somatostatin receptors that are expressed at the cell membrane of NET. High grade NET are more often detected by ^18^F-FDG PET/CT and thus FDG avidity can be an indicator of tumor aggressiveness [6].

MRI has been used in the characterization of NET but mostly on a morphological, qualitative basis with evaluation of tumor size, borders, signal intensity, absence or presence of cystic or necrotic components, and enhancement pattern. More advanced MR imaging techniques such as diffusion-weighted imaging (DWI) and more importantly, quantitative evaluation of apparent diffusion coefficient (ADC) mapping may have an added value. The correlation of ADC values and tumor cellularity or aggressiveness/prognosis has been investigated extensively in other tumor types (i.e. prostate adenocarcinoma [7] and astrocytic brain tumors [8]) where ADC values negatively correlate with tumor cellularity and aggressiveness. Numerous small studies indicate that similar findings may apply to NET but validation of these studies is still needed [9,10,11,12,13,14,15,16,17,18].

## Purpose

To investigate the possible correlation between average and minimum ADC values of NET on MRI and the histopathological WHO grade and to determine if ADC values may help differentiate between low (G1 and G2) and high (G3) grade NET.

## Materials and Methods

### Case selection

For this retrospective study we included patients that were presented at the Multidisciplinary Neuroendocrine Tumor Board. This tumor board is part of a collaborative NETwork that has been set up between nine regional hospitals and the Antwerp University Hospital.

Ethical approval for this study was obtained from the Institutional Review Board (EC nr. 18/43/491) and informed consent was waived due to the retrospective nature of the study.

Elelectronic medical files from patients with a known NET discussed on the tumor board between November 2017 and April 2019 were analyzed retrospectively by a senior radiology resident and radiology staff member in consensus. Only patients with available MRI (primary tumor or metastasis), lesion size larger or equal to 1 cm, and known WHO tumor grade were included (n = 47).

### Data collection and image analysis

First, the following clinical parameters were noted in an Excel worksheet: age, sex, primary tumor location, presence of metastasis, and WHO tumor grade (taken from pathology report).

Next, the technical parameters of the MRI exams were registered in the same worksheet (vendor, model, field strength, and employed b-values). This includes Siemens 1.5T (n = 29), Siemens 3T (n = 11), Philips 1.5T (n = 4), Philips 3T (1) and GE 1.5T (n = 2) with a b value sequence of 50-600-1000 sec/mm² and 0–1000 sec/mm² being the most frequently used.

**Figure 1:**
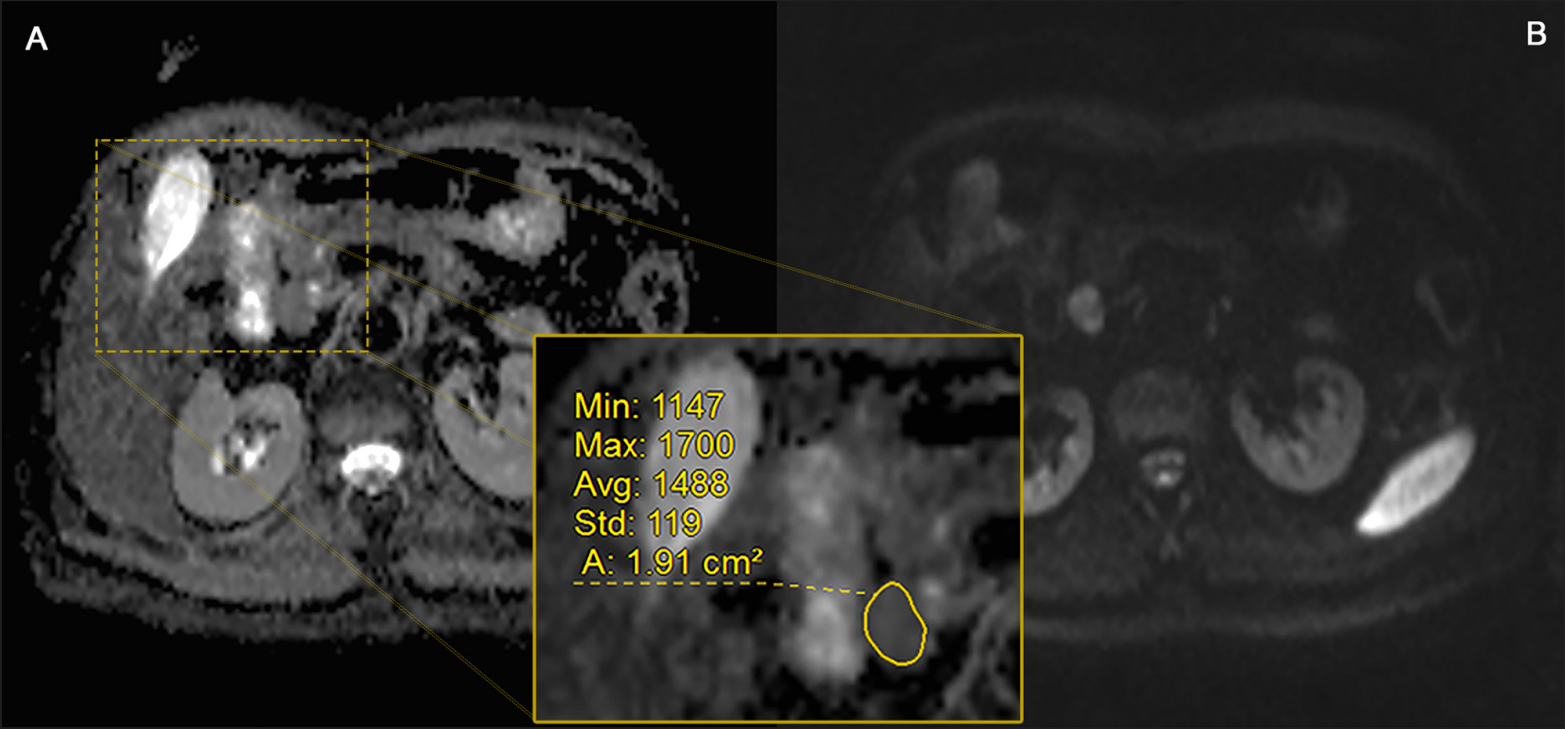
ROI placement (grade 1). Axial ADC map **(A)** and DWI b1000 **(B)** image with ROI placement along the borders of a WHO G1 lesion (resection proven paraduodenal metastasis) in a 52-year-old female.

**Figure 2:**
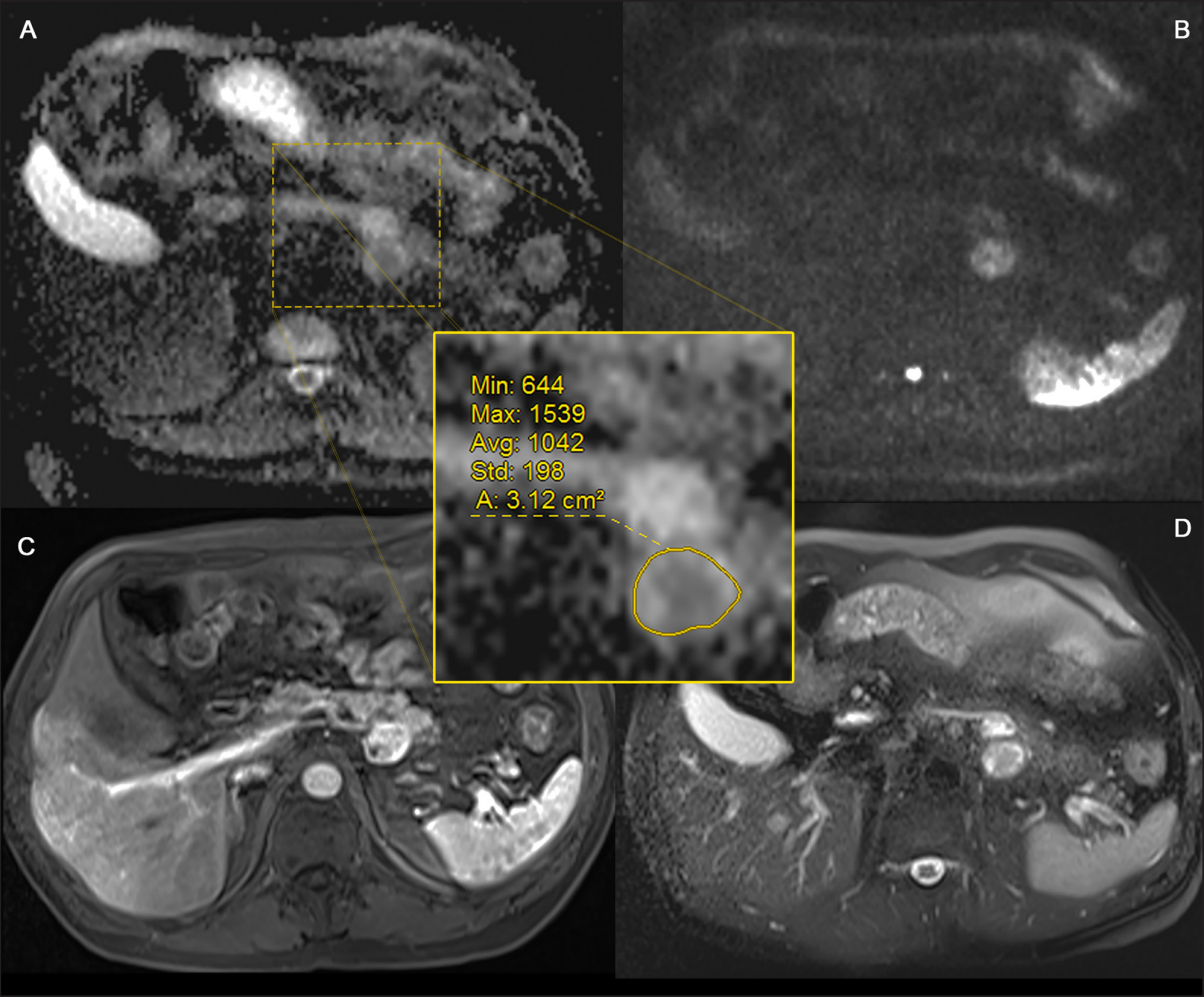
ROI placement (grade 2). Axial ADC map **(A)**, DWI b1000 **(B)**, arterial phase T1- weighted **(C)** and fat suppressed T2-weighted **(D)** images of a biopsy proven G2 NET in a 46-year-old male. ROI placement with exclusion of the outermost border of the lesion to avoid artefacts.

**Figure 3:**
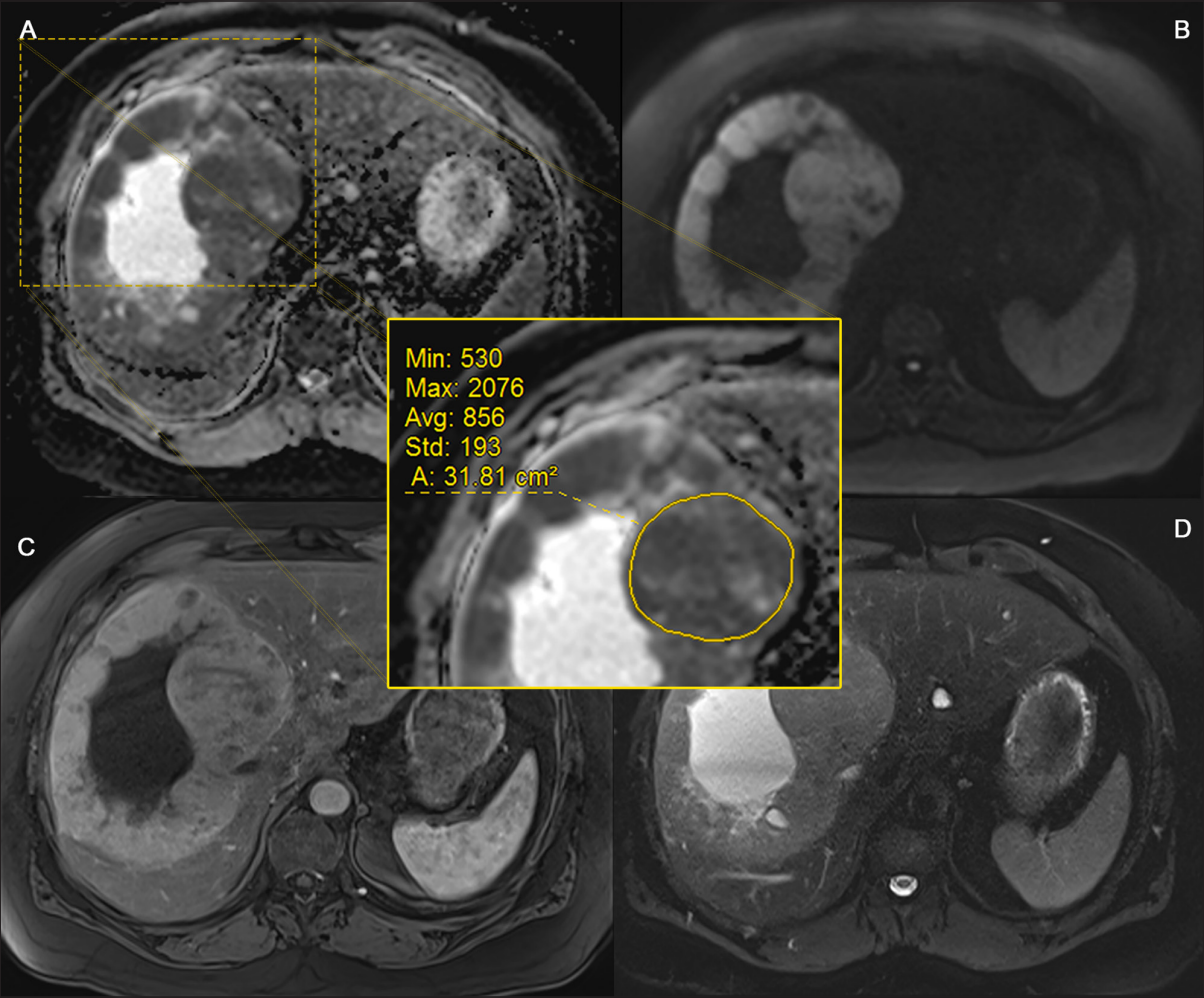
ROI placement (grade 3). Axial ADC map **(A)**, DWI b1000 **(B)**, arterial phase T1-weighted **(C)** and fat-suppressed T2-weighted **(D)** images of a biopsy proven G3 NET (primary tumor location = pancreas) in a 55-year-old male. Images show a large liver metastasis with cystic/necrotic centre: ROI placement in the border of the lesion, avoiding the cystic portion and the outermost edges.

The image analysis was done by a senior resident under supervision of a radiology staff member with more than ten years of experience in abdominal imaging. Measurements were performed on a picture archiving and communicaion system (PACS) workstation suited for clinical use (GE RIS/PACS). Average and minimum ADC values (avgADC; minADC) were measured by drawing a freehand region of interest (ROI) on either the center slice of the lesion or the level with the least artifacts (Figures 1, 2, 3). The T2-weighted images, DWI and contrast-enhanced T1-weighted images were used as a side by side reference. In case of multiple lesions (i.e. liver metastases) only the largest, most clearly delineated lesion was selected. The outermost border of the lesion and cystic or necrotic regions were omitted. In very large lesions with central necrosis, the ROI was drawn in the area with the highest intensity on the corresponding high b-value DWI series. In patients with MRI of both primary tumor and metastasis, separate ROIs were drawn for each and the corresponding ADC values were noted in the Excel worksheet.

Data were first sorted by WHO grade group and subsequently grouped together into low grade NET (WHO G1 and G2) and high grade NET (WHO G3) for further statistical analysis. When ADC values of both primary tumor and metastasis were available, the values chosen for analysis were those that matched the origin of the histopathologic specimen (i.e. biopsy of liver metastasis: ADC values of metastasis).

### Statistical analysis

Statistical analysis was conducted in SPSS Statistics (V25 – IBM). Non-parametric testing (Mann-Whitney-U) was used to compare differences in avgADC and minADC between low and high grade NET. Correlation between avgADC and minADC values and WHO grade was determined separately using R (V3.5.2 – The R Project for Statistical Computing) to compute Spearman’s rank correlation coefficient with 95% confidence intervals. Receiver-operating characteristic (ROC) analysis was performed using MedCalc for Windows (V15.1 – MedCalc Software, Ostend, Belgium) to estimate the area under the curve (AUC) with DeLong non-parametric method. Optimal cut-off values were determined according to the Youden index.

## Results

There were 47 patients included with a mean age of 59 (±12 SD; 30–88) with 24 males and 23 females. The final values that were used for analysis (after biopsy location matching) included 28 primary tumors and 19 metastases (Table 2). WHO grade distribution was 21 G1, 17 G2 and nine G3 tumors or 38 low grade (G1+G2) and nine high grade (G3) tumors. Metastatic disease was present in 47.6% of G1 tumors, in 76.5% of G2 tumors and in all G3 tumors.

**Table 2:** Measured lesion type and location.

Type	Organ	n

**Primary tumor**	Pancreas	24
	Small intestine	1
	Rectum	3
**Metastasis**	Liver	17
	Paraduodenal	1
	Mesenteric	1
**Total**		**47**

**Figure 4:**
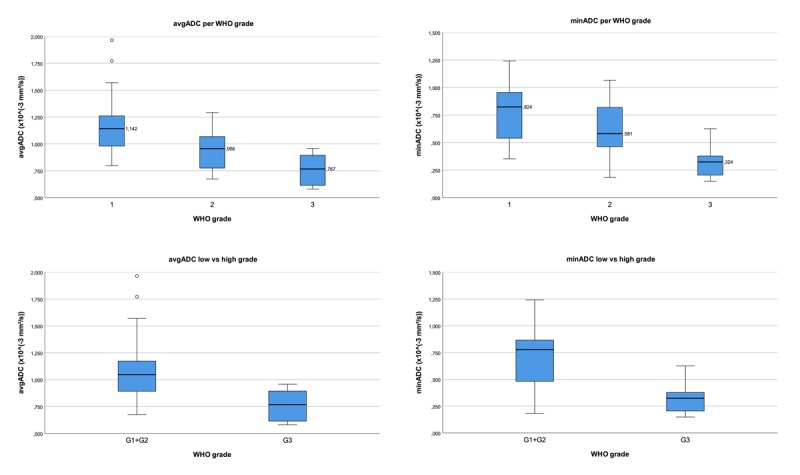
Boxplots of avgADC and minADC values per WHO grade. Boxplots illustrate the difference of avgADC and minADC values per WHO grade and low- vs. high-grade NET respectively. High-grade NET demonstrate lower ADC values than low-grade NET, but some overlap can be seen.

The distribution of avgADC and minADC values per WHO grade (separate and low vs high grade) are illustrated in Figure 4. The median avgADC value per grade group was G1: 1.142 (interquartile range (IQR) 0.347), G2: 0.956 (IQR 0.303) and G3: 0.767 (IQR 0.315). The median minADC value per grade group was G1: 0.824 (IQR 0.429), G2: 0.581 (IQR 0.371) and G3: 0.324 (IQR 0.244).

A significant difference was found between low-grade (G1+G2) and high-grade (G3) tumors (Mann-Whitney; avgADC: p < 0.001; minADC: p < 0.001). Separate, pairwise testing of WHO grades (Kruskal-Wallis) only showed a significant difference between G1 and G3 for both avgADC and minADC (p < 0.001). There was a moderate negative correlation between WHO-grade and avgADC/minADC (Spearman’s Rank; avgADC: –0.606; 95% CI [–0.773 to –0.384]; minADC: –0.581; 95% CI [–0.759 to –0.353]).

**Figure 5:**
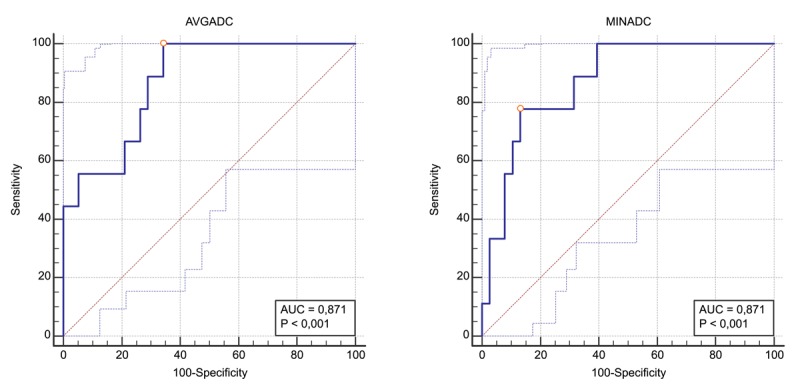
ROC curve analysis. ROC-curve analysis of avgADC and minADC demonstrates good accuracy of both values with calculated area under the curve (AUC) of 0.871 (p < 0.001).

ROC-curve analysis was performed for low grade vs high grade NET yielding an equal area under the curve (AUC) of 0.871 (95% CI [0.741; 0.951]) for both avgADC and minADC as illustrated in Figure 5. The optimal cut-off values were determined as ≤0.957 × 10^–3^ mm²/s (95% CI [≤0.935 to ≤0.957]; Youden index J 0.6579) for avgADC and ≤0.378 × 10^–3^ mm²/s (95% CI [≤0.324 to ≤0.626]; Youden index J 0.6462) for minADC. These cut-off values generate 100% sensitivity (SE) and 65.8% specificity (SP) for avgADC and 77.8% SE and 86.8% SP for minADC. The corresponding positive and negative predictive values (PPV; NPV) are 40.9% PPV and 100% NPV for avgADC and 58.3% PPV and 94.3% NPV for minADC.

## Discussion

Treatment strategy for NET is related to the histological tumor grade and more specific the differentiation between low grade (WHO G1 and G2) and high grade (G3) tumors [11]. Low grade NET can be treated with surgical resection and/or targeted therapy for example somatostatin analogs, receptor targeted radionuclide agents, bevacizumab, sunitinib, and everolimus [3,6]. High grade NET are treated with platinum-based chemotherapy. Alternative treatment strategies in inoperable patients include radiofrequency ablation, transarterial chemoembolization, and radioembolization. Both treatment strategy and prognosis are strongly dependent on histological grade with a poorer prognosis and higher metastatic rate for G3 tumors [9,11]. The possibility to predict tumor grade on a noninvasive basis and without ionizing radiation would certainly be advantageous. Early risk stratification can help with disease management and prevents treatment delay.

The purpose of this study was to evaluate the correlation of ADC values on MRI with the histopathologicaly based WHO grading. Our results show a significant difference in both avgADC and minADC values between low grade (G1+G2) and high grade (G3) NETs. These findings are in line with other studies that compared G1+G2 vs G3 NET [9,11,13,14,17,18]. We chose to compare G1+G2 vs G3 because of the different treatment and prognosis of these groups as mentioned above. However, some studies have chosen to compare G1 vs. G2+G3 NET [10,12] or compare each group separately [13,14,16,19]. When comparing differences in avgADC between separate WHO grade groups we could only find a significant difference between G1 and G3 (p < 0.001) but not between G1 and G2 (p = 0.058) or G2 and G3 (p = 0.117). This was also the case for Pereira et al. [16] (G2 vs. G3) and Besa et al. [9] (G1 vs. G2 and G2 vs. G3). Lotfalizadeh et al. [14] and Kulali et al. [13] found significant differences between all separate grade groups. Few studies include the minADC values like in our study [9,14]. Besa et al. [9] found similar minADC values whereas Lotfalizadeh et al. [14] found higher minADC values (Table 3). We found a significant difference in minADC between low-grade (G1+G2) and high-grade (G3) NET and between G1 versus G3 NET (p < 0.001) when comparing groups separately. There was no significant difference in minADC between G1 and G2 (p = 0.110) and G2 and G3 (p = 0.089), similar to what Besa et al. reported [9].

**Table 3:** Comparison of avgADC and minADC values with previous studies.

Author	n NET (G1, G2, G3)	avgADC (×10^–3^ mm^2^/s) mean ± SD			minADC (×10^–3^ mm^2^/s) mean ± SD		

		G1	G2	G3	G4	G5	G6

Mebis et al. 2020	47 (21, 17, 9)	1.18 ± 0.31	0.95 ± 0.18	0.76 ± 0.15	0.79 ± 0.25	0.60 ± 0.24	0.34 ± 0.15
		1.08 ± 0.28			0.71 ± 0.26		
Besa et al. 2016	48 (25, 16, 7)	1.47 ± 0.63	1.27 ± 0.63	0.87 ± 0.43	0.84 ± 0.55	0.50 ± 0.48	0.27 ± 0.41
Guo et al. 2017	59 (34, 13, 12)	1.09 ± 0.13		0.85 ± 0.23		/	
De Robertis et al. 2017	55 (31, 20, 4)	1.29 ± 0.47	1.09 ± 0.28			/	
Min et al. 2018	63 (3, 27, 33)	1.06* (1.05–1.19)	0.82* (5.6–1.42)	0.59* (0.26–0.95)		/	
Jang et al. 2014	34 (20, 14 G2+G3)	1.48° (0.91–2.51)	1.04 (0.48–1.92)°			/	
Lotfalizadeh et al. 2016	108 (55, 42, 11)	2.13 ± 0.70	1.78 ± 0.72	0.86 ± 0.22	1.52 ± 0.59	1.33 ± 0.49	0.78 ± 0.22
Pereira et al. 2015	22 (15, 4, 3)	1.28 ± 0.27^#^	0.89 ± 0.39^#^	0.73 ± 0.23^#^		/	
Wang et al. 2011	18 (12 G1+G2, 6)	1.75 ± 0.53		1.00 ± 0.19		/	
Kulali et al. 2017	30 (9, 10, 11)	2.32 ± 0.15	1.29 ± 0.15	0.88 ± 0.15		/	
Kim et al. 2013	39 (24, 12, 3)	1.60 ± 0.41	1.24 ± 0.13	/		/	

* median (range); ° mean (range); ^#^ mean ± SE (standard error).

Min et al. [15] did not compare differences between groups but found a significant (p < 0.001) moderate negative correlation between avgADC and WHO grade (–0.57), in concordance with the negative correlation of –0.61 we found and the negative correlation of –0.55 Lotfalizadeh et al. reported [14]. Besa et al. [9] found a significant but weaker negative correlation of –0.33. Others reported a negative correlation between avgADC and Ki67-index (Guo et al. [11]: –0.41, Wang et al. [17]: –0.70). The avgADC and minADC values we found per WHO grade were similar to the values found by previous studies and are summarized in Table 3. Only the values acquired by Lotfalizadeh et al. [14] are markedly higher. The observed differences are probably multifactorial with not only tumor heterogeneity, relatively small sample size, technical differences (i.e. field strength, b-values), and measuring methods (i.e. center slice or volumetric).

Expanding on the abovementioned technical differences, it is interesting to see that despite the different vendors, field-strengths, and b-values that were used, the acquired ADC values were similar to what most other authors found (some of them using only one device and some using multiple devices). We assume that the technical differences cause added noise in the data but are too small to have an effect on the final results. A direct comparison between vendors, field-strengths, and b-values would be interesting but is hard to achieve given the small sample size. Achieving similar results with different machines might facilitate the implementation of the acquired values in daily practice.

The decrease in ADC values in high-grade tumors can be attributed to the increased cellularity with decreased extracellular space and cytoplasmic volume (high nucleus-to-cytoplasm ratio) restricting the movement of water molecules [16,17]. Other factors such as fibrosis can also contribute to a lower ADC and might be an explanation for the lower ADC values in some low grade, well-differentiated tumors [17].

We found a good accuracy of avgADC and minADC in predicting G3 vs G1+G2 NET with an AUC of 0.871 for both. Other studies found similar values: Besa et al. [9]: 0.80 and 0.76, Guo et al. [11]: 0.90 and Lotfalizadeh et al. [14]: 0.96 and 0.83. A meta-analysis performed by Zong et al. [18] showed a summary AUC of 0.94 for predicting G3 from G1+G2 tumors. The ‘optimal’ cut-off values we acquired (according the Youden index) were ≤0.957 × 10^–3^ mm²/s for avgADC and ≤0.378 × 10^–3^ mm²/s for minADC. A comparison of these values and their associated SE, SP, PPV, and NPV with results of other authors is summarized in Table 4. Our cut-off value for meanADC was similar to the one Guo et al. [11] found (≤0.950 × 10^–3^ mm²/s) whereas other studies reported cut-off values that are slightly higher [9,13,14]. The SE and NPV for avgADC (both 100%) in our study were comparable but SP and PPV are lower. Only one study [9] reported a cut-off value for minADC (≤0.150 ×10^–3^ mm²/s) which is markedly lower than the value we found (≤0.378 × 10^–3^ mm²/s) and yielded lower SE, SP, PPV, and NPV. This difference in minADC value could be explained by the fact that the minimum value is only a single value that is more prone to artefacts and ROI placement than an average value.

**Table 4:** Comparison of cut-off values and diagnostic performance.

Author	avgADC					minADC				

	Cut-off ×10^–3^ mm^2^/s	SE %	SP %	PPV %	NPV %	Cut-off ×10^–3^ mm^2^/s	SE %	SP %	PPV %	NPV %

Mebis et al. 2020	≤0.957	100	65.79	40.91	100	≤0.378	77.78	86.84	58.33	94.29
Besa et al. 2016	≤1.24	100	84.21	*59.77*	*100*	≤0.15	50.00	84.21	*42.62*	*87.78*
Guo et al. 2017	≤0.950	72.3	91.6	*66.88*	*93.38*			/		
Lotfalizadeh et al. 2016	≤1.19	100	92	*74.57*	*100*			/		
Kulali et al. 2017	≤1.20	100	84.20	*59.75*	*100*			/		
	PPV and NPV values in italic are calculated post-hoc (with estimated prevalence of 19%)									

Average ADC value (avgADC), minimum ADC value (minADC), sensitivity (SE), specificity (SP), positive predictive value (PPV), negative predictive value (NPV).

We acknowledge several limitations of our study. First, the retrospective nature of the study comes with a number of disadvantages including selection bias, lack of standardization of imaging techniques and equipment, and possible influence of already initialized treatment. Secondly, ROI placement was performed in consensus and not repeated meaning intra- and interobserver agreement could not be tested. Image analysis was not entirely blinded for WHO-grade (as patients reviewed were discussed on the tumor board). Thirdly, although comparable to other studies, the sample size of G3 NET was relatively small. The latter can both be explained by the rarity of G3 tumors and the fact that during initial selection it became evident that most G3 patients did not have an MRI exam but had an ^18^F-FDG-PET-CT followed by debulking or resection, often after neoadjuvant systemic treatment. Consequently G3 sample size was also too small to compare differences between G3 NET and G3 NEC as determined by the WHO 2017 criteria. Histopathologic grade was derived from the multidisciplinary staff report or pathology report without extra information about the cellularity or possible fibrosis, which could explain some of the heterogeneity (as reported by [17]). Ki-67-index was often reported as a range instead of a single percentage making it unreliable for stratification. Histopathologic diagnosis was based on both surgical resection and biopsy, the latter being less reliable. We did not compare the ADC values to normal tissue/negative controls, however in some cases this would not have been possible (i.e. diffuse liver metastasis). Finally, the defined cut-off values and their corresponding predictive statistics should be interpreted with caution because of the small sample size and the unknown prevalence of G3 NET in the general population. The prevalence used to calculate PPV and NPV values is based on the prevalence of G3 NET in our study (19%), which is similar to the percentage found in larger population studies [2,4] but this still needs to be verified.

## Conclusion

We are able to confirm the assumed negative correlation between ADC values and tumor grade and found a significant difference of avgADC and minADC between grouped low-grade (G1+G2) and high-grade (G3) NET. The findings of our study are in line with previous studies despite the aforementioned technical differences, which might facilitate the implementation of the achieved values in daily practice.
